# Geometric Simulation Approach for Grading and Assessing the Thermostability of CALBs

**DOI:** 10.1155/2016/4101059

**Published:** 2016-03-31

**Authors:** B. Senthilkumar, D. Meshachpaul, R. Rajasekaran

**Affiliations:** Computational Biology Lab, Department of Biotechnology, School of Bio Sciences and Technology, VIT University, Vellore, Tamil Nadu 632014, India

## Abstract

*Candida antarctica* lipase B (CALB) is a known stable and highly active enzyme used widely in biodiesel synthesis. In this work, the stability of native (4K6G) and mutant (4K5Q) CALB was studied through various structural parameters using conformational sampling approach. The contours of polar surface area and surface area of mutant CALB were 11357.67 Å^2^ and 30007.4 Å^2^, respectively, showing an enhanced stability compared to native CALB with a statistically significant *P* value of < 0.0001. Moreover, simulated thermal denaturation of CALB, a process involving dilution of hydrogen bond, significantly shielded against different intervals of energy application in mutant CALB revealing its augmentation of structural rigidity against native CALB. Finally, computational docking analysis showed an increase in the binding affinity of CALB and its substrate (triglyceride) in mutant CALB with Atomic Contact Energy (ACE) of −91.23 kcal/mol compared to native CALB (ACE of −70.3 kcal/mol). The computational observations proposed that the use of mutant CALB (4K5Q) could serve as a best template for production of biodiesel in the future. Additionally, it can also be used as a template to identify efficient thermostable lipases through further mutations.

## 1. Introduction

Lipases (EC 3.1.1.3, triacylglycerol lipase) exist as proficient catalysts of high demand in varied fields. They are considered as remarkable molecular devices that determine patterns of chemical transformations like hydrolyzation, acidolysis, alcoholysis, aminolysis, esterification, interesterification, and transesterification [1]. Psychrophilic lipases are used widely in various industrial applications such as pharmaceutical productions, food industry, detergents, waste treatment, and bioremediation and recently in biodiesel production for their lipolytic activity. Microbes serve as a highly preferred source for wide variety of enzymes that are used in industrial firms. Industrial enzymes produced are widely adapted from yeast compared to bacteria, as yeast is easy to handle and grows in large scale [2].

*Candida antarctica* lipase B (CALB) obtained from the yeast* Candida antarctica* belongs to the enzyme class hydrolases, which specifically react with carboxylic ester bonds.* Candida antarctica* exhibits two forms of lipases (A and B) with varied physiochemical properties. Lipase B (CALB) is widely used in ester synthesis, desymmetrization, production of laboratory peracids, and other commercial products, as it is small, acidic, and less thermostable than lipase A [3]. CALB is an *α*/*β* globular protein with 317 amino acids weighing 33,273 Da, consisting of a conserved region with eight twisted *β* strands and an *α*-helix arranged on either side. CALB differs from other lipases as it is not shown to undergo interfacial activation, a phenomenon where lipase undergoes a conformational change associated with increased enzymatic activity when adsorbed onto a lipid surface [1, 4]. CALB is a serine hydrolase, consisting of Ser-His-Asp catalytic triad with serine acting as a nucleophile [5]. It is well known in industrial field for its chemical property involving the hydrolysis of triacylglycerols into fatty acids, diacylglycerol, monoacylglycerol, and glycerol [6]. It is characterized for its stereoselectivity, thermal stability, and activity, making it a common choice of enzyme for organic synthesis reactions and a competent biocatalyst for industrial applications. Industrial importance of CALB is due to its high enantioselectivity against secondary alcohols and its ability to survive high temperatures in organic solvents. Reduction in binding pocket size of CALB enables it to act highly on straight chain acids containing 5 to 12 carbon atoms [7].

CALB has advanced itself into the field of biodiesel production, as it possesses high stability and activity in the presence of alcohols. The industrial processing units involve high temperatures; hence, to cope with these challenges, improvement of a thermostable CALB with good enzyme activity is highly recommended. In this work, native CALB and mutant CALB were studied computationally using various structural stability features such as polar surface area (PSA), surface area (SA), energy distribution, hydrogen bond dilution, and docking to grade and assess their thermostability and functional activity. Experimental studies supported the reliability of CALB as a stable and active lipase but not much of computational works to support its stability are found. This study aims at computational prediction of thermostable CALB that could be used as a template for the synthesis of biodiesel and also an efficient model for appending further mutations to increase its strength in the future. In addition, the effect of conserved pattern in structural events using conformational sampling through normal mode-based geometric simulation showed reliable results as done by classical molecular dynamics comparatively [8]. Thus, this work aided in the computational understanding of CALB thermostable characterization and also instigating new development of efficient and robust computational method that could reduce time and laborious work.

## 2. Materials and Methods 

### 2.1. Dataset

The steric constrained crystal structures of 4K6G (native) CALB and 4K5Q (mutant) CALB along with their related files were collected from Protein Data Bank (PDB) at http://www.rcsb.org/ [9]. The structural files were analyzed computationally for various physiochemical properties.

### 2.2. Structural Refinement and Conformational Sampling

The PDB structures of both native CALB and mutant CALB were structurally refined by KOBAMIN [10], which works on knowledge-based potential of mean force minimization, following which the structures were stereochemically corrected using MESHI. These energy minimized structures were scrutinized and utilized further in cluster analysis and single model analysis.

For cluster analysis, NMSim, a multiscale modeling program for protein conformational transitions, was used. Based on geometric simulations, the process was carried in three steps: (i) coarse grain of protein using FIRST software, (ii) normal mode analysis of rigid clusters, and (iii) geometric simulations (NMSim). Stereochemical ensemble of protein conformations along with their corresponding conformational trajectory and data plots were distinguished via structural analysis [11]. The calculations involving parameters such as polar surface area (PSA) and surface area (SA) of obtained conformational trajectories were evaluated via Vega ZZ, a molecular modeling program [12]. The variations in their values explained solvent accessibility and surface level interactions of the given protein.

The trajectory files of each protein were subjected to Bayesian Analysis Conformation Hunt (BACH), which discriminated the best model through BACHSCORE. The BACH program compares the given protein with similar protein structures obtained from various databases via global distant testing and atom energy scores. The program displayed protein conformations and their corresponding energy score as output; thus the one with least energy was predicted to be the best conformation (global minima) correspondingly [13]. This best conformation was used in single model analysis to compute effect of stability and functional activity by hydrogen bond dilution method and docking technique, respectively.

### 2.3. Hydrogen Bond Dilution

Various studies depicted the vital role played by hydrogen bonds in improved protein stability. The formation of stable hydrogen bonds relies on distance and angular distribution of both acceptor and donor molecules. On introduction of thermal energy, the hydrogen bonds begin to lose hold and break apart based on their distance, energy supplied, and their positions. MSU ProFlex (formerly called FIRST), a computational tool embedded in the program StoneHinge, was used to identify rigid and flexible regions of known proteins. StoneHingeP and StoneHingeD modules incorporated in StoneHinge program aided in the prediction of flexible hinge using ProFlex and its rigidity by DomDecomp analysis. Both analyses together helped in identifying both flexible and rigid regions of the given protein, CALB, in this study [14].

### 2.4. Docking Analysis

The role of receptor-ligand interaction helps to study the conceptual biology of protein function via docking analysis. A geometry-based molecular docking algorithm proposed by Schneidman-Duhovny et al. [15] was used for docking CALB with the substrate triglyceride. Triglyceride exists as a potent substrate for CALB both* in vitro* and* in vivo*. Hence, the SMILES string of molecule triglyceride was obtained from PubChem (http://pubchem.ncbi.nlm.nih.gov/) and its 3D structure was generated using CORINA (http://www.molecular-networks.com) [16]. Docking aids in identifying the transformation of one molecule at which other molecules could fit in devoid of any steric changes [17, 18]. This depended not only on the geometric form of molecules but also on their complex stability. The program received both receptor and ligand molecules as PDB files, thus performing algorithmic calculations based on three stages: molecular shape representation, surface patch matching, and ranked scoring. The program rendered an output based on their shape complementarity, interface shape, and sizes of the molecules, and they were given as solutions along with their geometric score, desolvation energy, area size, and the docked PDB file. This helped to read the best template among mutants with high enzymatic activity [15]. The docked molecules were visualized using PyMOL, a molecular modeling package (The PyMOL Molecular Graphics System, Version 1.7.4 Schrödinger, LLC).

### 2.5. Statistical Analysis

The nonparametric values obtained via molecular simulation program were analyzed for statistical significance. Mann-Whitney test, also known as rank-sum test, was utilized for the comparison of nonparametric unpaired groups [19]. Thereby, using MS-Excel, the nonparametric mean values of native CALB and mutant CALB were compared simultaneously. The significance of the parameters was calculated by two-tail probability value (*P* value) and *P* < 0.05 was considered statistically significant.

## 3. Results and Discussion

**Figure 1:**
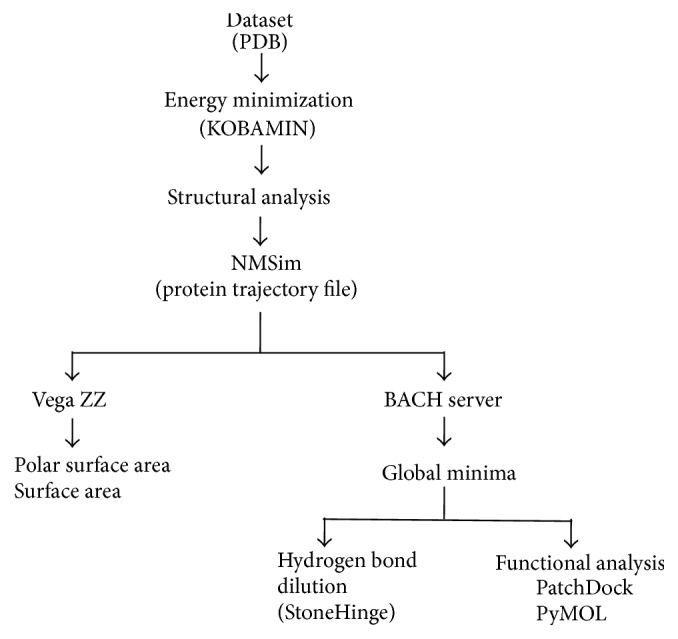
Flowchart illustrated the methodology and the various bioinformatics tools used in this study.

Active site is said to play a vital role in enzymatic catalysis of a protein, thus aiding in its kinetic stability. The parameters constituting stability of native CALB (4K6G) and its most thermostatic mutant D223G/L278M (4K5Q) CALB protein [8] were evaluated based on the following: (a) polar surface area, (b) solvent accessibility, (c) energy distribution, (d) hydrogen bond dilution, and (e) binding affinity (Figure 1). These physiochemical parameters aided in the evaluation of structural stability and functionality of CALB, through conformational sampling.

### 3.1. Retrieval of Native and Mutant Structure of CALB

CALB belongs to *α*/*β* hydrolase class protein, globally known for its dual role in ester hydrolysis as well as in synthesis [4]. High resolution crystal structures (<1.5 Å) of native CALB and mutant CALB were collected from Protein Data Bank (PDB) for analyzing various physiochemical parameters contextual to thermostability. In this study, conformational sampling was utilized, which yielded an ensemble of protein conformations for cluster analysis. Subsequently, energetically best model among the conformations was selected for analyzing hydrogen bond dilution and binding affinity.

### 3.2. Degree of Solvability and Surface Accessibility of CALB

CALB exists as functionally known biocatalyst associated with the hydrolysis of water and esterification of various organic solvents [20]; hence, the study of interactions involving polar molecules in a solvable environment gains importance. CALB exhibited molecular heterogeneity, the ability of a molecule over time to assume several different conformations with trivial difference in stability, an innate property of all large biomolecules [21]. The cluster analysis was performed with the ensemble conformations obtained through the simulatory tool NMSim, using Vega ZZ program. The major parameters investigated for viewing the stability of a protein were RMSD and RMSF. Both these parameters showed high rigidity in mutant CALB compared to native CALB, similar to that of classic molecular dynamics studies [8], thereby demonstrating the eminence of structural variations in protein CALB stability and reliability of conformational sampling. With the reasoning of structural deviations for stability, stereochemical features of CALB at surface level, such as PSA and SA, were further analyzed.

**Figure 2:**
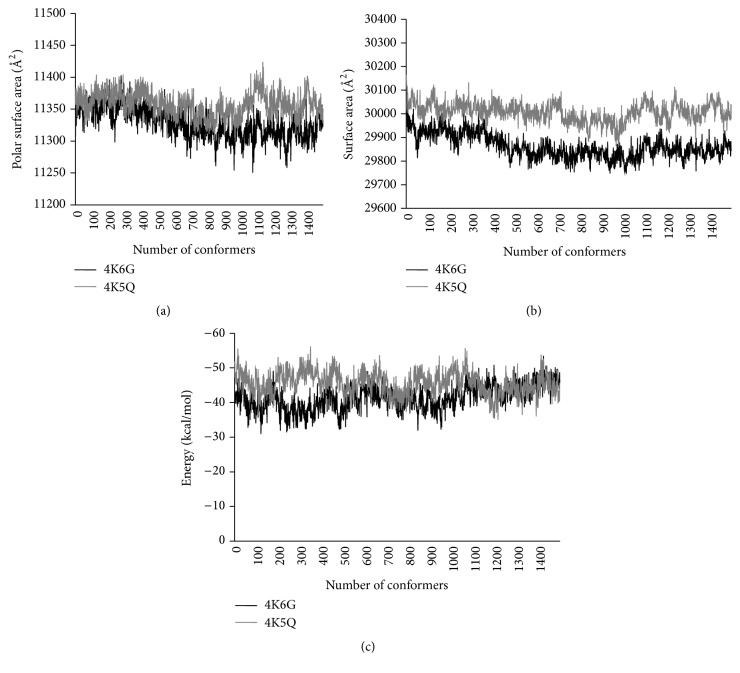
Geometrical observables of native (4K6G) CALB and mutant (4K5Q) CALB. (a) Polar surface area. (b) Surface area. (c) Energy levels calibrated by knowledge-based potential.

The dissolution of a protein in water depends on two interactions: (1) the strength with which a protein molecule associates with the solvent and (2) how tightly the compound was bound to its own crystal lattice. PSA remains to be a part of van der Waals or solvent-accessible surface and their sum of surfaces that contained both polar (O, S, N, P, and H not bonded to C) and apolar atoms (C and H bonded to C) in the given molecule. PSA was found to be increased along with the count of hydrogen bonds formed between protein surface and water, thereby constituting increased thermostability [22]. The PSA mean data of the mutant CALB (11357.67 Å^2^) was found to be higher than that of the native CALB (11328.08 Å^2^) with a statistically significant *P* value (Mann-Whitney test, *P* value < 0.0001). Thus increased PSA was found to increase the hydrogen bonds relatively, thereby defining stability of the mutant CALB (4K5Q) as shown in Figure 2(a).

The ability of a protein molecule to interact with its solvent counterpart highly depends on the presence or absence of hydrophobic residues on its surface. As per previous studies, CALB was found capable of hydrolyzing soluble as well as insoluble substrates, with high level activity at water-lipid interfaces [23]. The ratio of buried nonpolar surface area to total nonpolar surface area of a protein was identified as hydrophobicity. Hydrophobic forces played a vital role in regard to thermostability and molecular folding of a protein [24, 25]. Whereas the chemical reactions related to CALB majorly involved solvents in a solvable environment, exposure of polar residues on the surface was considered functionally important. The mean surface area of the mutant CALB (30007.4 Å^2^) showed an increase compared to that of native CALB (29862.5 Å^2^) with a statistically significant *P* value less than 0.001 (Figure 2(b)). The results obtained might forecast the role polar residues played in sustaining functional aspect of mutant CALB, that is, activity in the presence of solvents such as alcohols.

### 3.3. Energy Allocation

The steric conformers simulated through NMSim were subjected for calculating energy contour using BACH program. The BACH energy was calculated using effective pairwise residue-residue interactions along with single residue solvation property of the molecule. The graphical representation of energy profiles showed large number of mutant CALB conformers (mean energy: −45.57 kcal/mol) possessing low energy profiles compared to native CALB (mean energy: −41.53 kcal/mol) with statistically significant *P* value less than 0.0001, respectively (Figure 2(c)). Thereby a decrease in energy profile might be a corresponding factor for improved functionality and stability of mutant CALB compared to native CALB [13]. Among given conformations, the conformer with least energy also known as global minima was selected for further studies involving hydrogen bonds and functionality of CALB.

### 3.4. Hydrogen Bond Dilution of CALB

Among various stability enhancing interactions, hydrogen bonds are said to be an important parameter for stability. The global minima conformer of both native CALB and mutant CALB was selected to analyze hydrogen bond dilution, which may enlighten the understanding of mutant stability stereochemically. At intermittent state of energy levels, hydrogen bonds get diluted based on their steric properties, resulting in an unfolded protein structure, thus explaining the stability of a protein. The unfolding of protein structure resulted in loss of secondary structure and stability, due to breaking up of hydrogen bonds and salt bridges. Thermal denaturation was functionally implicated for the dilution of hydrogen bonds, thus resulting in breakage of weakest bonds followed by stronger ones. This in turn aided in the discovery of flexible and rigid areas of a given structural protein [14]. The hydrogen dilution pattern of native CALB and mutant CALB was analyzed using MSU ProFlex; the data obtained enriched us more of the role of hydrogen bonds in stability of CALB. Moreover, the results clearly stated that energy required to break a hydrogen bond was directly proportional to the strength of hydrogen bond; that is, the stronger the bonding the higher energy requirement [26, 27]. The data depicting the number of bonds broken after each energy cycle (Table 1) predicted the strength and steric stability of remaining hydrogen bonds in maintaining overall structural flexibility and rigidity of CALB.

**Figure 3:**
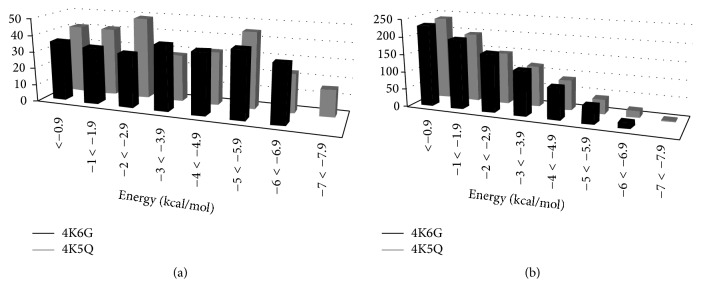
Comparative graphical representation of (a) the number of hydrogen bonds broken and (b) the number of hydrogen bonds remaining to be broken, at different energy levels for native (4K6G) CALB and mutant (4K5Q) CALB.

Three stable hydrogen bonds remained unbroken in mutant CALB after supplying energy of more than −8 kcal/mol. In contrast, complete hydrogen bond dilution took place before supplying energy more than −7 kcal/mol in native CALB (Figure 3). The data also showed that a significant number of bonds were broken both in native CALB (40) and in mutant CALB (45) between energy levels of −5 kcal/mol and −5.9 kcal/mol comparatively. In addition to current observations earlier studies too supported the fact that hydrogen bonding increases stability and rigidity of CALB [22].

### 3.5. Binding Affinity of CALB

**Figure 4:**
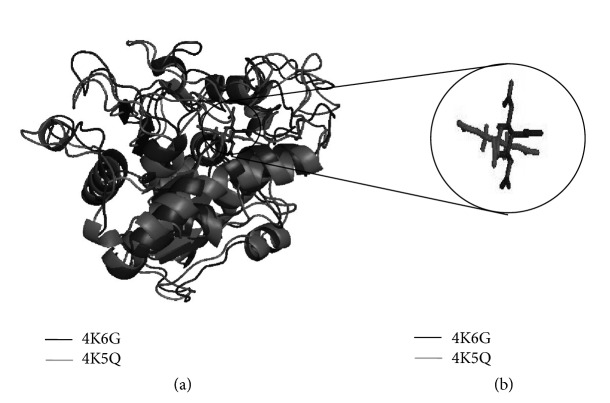
An illustration of the docked enzymes native CALB (4K6G) and mutant CALB (4K5Q) bound to their ligand triglyceride: (a) superimposed structure of the docked CALB and (b) the orientation of the ligand triglyceride at the active site 130.

The functional enzymatic efficiency and binding capacity of both native CALB and mutant CALB with their substrate triglyceride were studied using PatchDock. The program expressed the results as Atomic Contact Energy (ACE). The ACE of the mutant CALB (−91.23 kcal/mol) showed high binding affinity compared to that of native CALB (−70.3 kcal/mol) at active site 130 [25]. The result showed a change induced in native CALB due to mutation could be a potent reason for the increase in binding efficiency of its mutant counterpart. The alteration in symmetric conformation of superimposed docked complexes of both native CALB and mutant CALB was illustrated in Figure 4, representing the point at which CALB was appended with substrate triglyceride. The ligand molecules were bound in such a manner that they face each other at a single point.

## 4. Conclusions

CALB exists as highly industrial oriented enzyme in present age and is known for its various functional aspects predominantly in biodiesel production. The native CALB and its corresponding mutant CALB were analyzed computationally; as biodiesel production involves hydrolysis of alcohols by CALB, an in-depth analysis of parameters like solubility and surface accessibility gains importance in this study. Cluster analysis was carried out using conformation sampling method in which PSA and SA of mutant CALB showed their significance in solvent accessibility, thereby defining functional activity of CALB. Hydrogen bond dilution showed lack of stable hydrogen bonds in native CALB beyond energy level of −7 kcal/mol, whereas mutant CALB showed a presence of 22 bonds to be thermally denatured at a supply of more than −7 kcal/mol of energy. In addition, docking analysis performed using PatchDock showed a difference in ACE between the mutant CALB (−91.23 kcal/mol) and native CALB (−70.3 kcal/mol), representing their functional enzymatic activity, which depicted an increase in mutant binding affinity. The characterization and production of thermostable lipase with selective activity became a great demand in the field of biotechnology [28]. This study clearly states that the use of CALB mutant as a stable template could be a reliable source for enzymatic processes in biotechnology firm. It could also be utilized as a starter template for introducing mutations and designing high thermostable CALBs in the future. Moreover, the application of conformational sampling showed a lucid level of similarity with classic molecular dynamics, thus supporting the use of conformational sampling in simulation analysis aiding in timely results.

## Figures and Tables

**Table 1:** Hydrogen bond dilution data of native (4K6G) and mutant (4K5Q) CALB showing the number of bonds broken and the number of bonds remaining to be broken at different energy profile.

SI number	Energy supplied (kcal/mol)	4K6G		4K5Q	
		Bonds broken	Bonds remaining	Bonds broken	Bonds remaining
1	<−0.9	36	233	41	237
2	−1 < −1.9	34	199	41	196
3	−2 < −2.9	32	167	49	147
4	−3 < −3.9	39	128	28	119
5	−4 < −4.9	37	91	32	87
6	−5 < −5.9	40	51	45	42
7	−6 < −6.9	34	17	23	19
8	−7 < −7.9	0	0	16	3
